# A Ferroptosis-Related lncRNA Model to Enhance the Predicted Value of Cervical Cancer

**DOI:** 10.1155/2022/6080049

**Published:** 2022-02-08

**Authors:** Zhaojing Jiang, Jingyu Li, Wenqing Feng, Yujie Sun, Junguo Bu

**Affiliations:** ^1^Department of Radiotherapy, Zhujiang Hospital, Southern Medical University, Guangzhou, China; ^2^Department of Pathology, Zhujiang Hospital, Southern Medical University, Guangzhou, China; ^3^Department of Oncology, Zhujiang Hospital, Southern Medical University, Guangzhou, China; ^4^Department of Oncology, Guangdong Second Provincial General Hospital, Guangzhou, China

## Abstract

**Background:**

Cervical cancer (CC) is a common gynecological malignant tumor. Ferroptosis is a new type of programmed cell death, which plays a crucial part in cancer. However, current knowledge regarding ferroptosis-related long noncoding RNAs (lncRNAs) in CC is still limited. Therefore, our aim is to identify ferroptosis-related lncRNAs, build a steady prediction model, and improve the prediction value of CC.

**Methods:**

We obtained RNA expression and ferroptosis-related gene data of female CC patients from TCGA and FerrDb databases, respectively. Then, the ferroptosis-related lncRNAs were obtained by the limma R package and Cytoscape 3.7.1. We constructed the prediction model by Cox regression analysis. Finally, the prediction model was verified by the median risk score, Kaplan–Meier analysis, the time-dependent receiver operating characteristic (ROC) curve, clinical features, and immunoinfiltration analysis.

**Results:**

We acquired 1393 ferroptosis-related lncRNAs. The ferroptosis-related lncRNA signature was obtained by multivariate Cox regression analysis, and the patients were divided into a high-risk group and a low-risk group. The prognosis of the high-risk group was worse than that of the low-risk group. We found that the risk score can be used as an independent prognostic index by multivariate Cox regression analysis. The area under the time-dependent ROC curve reached 0.847 at 1 year, 0.906 at 2 years, 0.807 at 3 years, and 0.724 at 5 years in the training cohort. Principal component analysis showed that the diffusion directions of the two groups were different. Gene set enrichment analysis indicated that lncRNAs of two groups may be involved in tumorigenesis. Further analysis showed that high-risk groups were related to immune-related pathways. Ferroptosis-related lncRNAs are related to the proportion of tumor-infiltrating immune cells in CC.

**Conclusion:**

We have constructed a ferroptosis-related lncRNA prediction model. The prognostic model had important clinical significance, including improving the predictive value and guiding the individualized treatment of CC patients.

## 1. Introduction

Cervical cancer (CC) is a serious threat to women's health [1]. Many people around the world die of this cancer every year [2]. Human papillomavirus (HPV) infection is an essential factor for developing CC [3]. The incidence of CC has dropped by 40% to 50% in recent years, due to the wide application of early cervical cancer screening and advances in surgical, radiotherapy, and chemotherapy treatments [4]. Although the popularity of the HPV vaccine has reduced the number and mortality of CC patients, many women still suffer from CC [5, 6]. Therefore, it is imperative to find an ideal clinical model or accurate prognostic biomarkers that instruct the treatment of CC.

In the past few decades, the research on ferroptosis of tumors has increased rapidly. Different from apoptosis and autophagy, this is a new mode of nonapoptotic cell death that relies on the accumulation of reactive oxygen species (ROS) in an iron-dependent manner [7]. At present, many studies have shown that ferroptosis plays a vital role in mediating tumor development and drug resistance [8]. Ubellacker et al. [9] reported that melanoma cells in lymph have a higher ability to metastasize due to their resistance to ferroptosis. In tumor treatment, chemotherapy can induce ferroptosis of cancer cells, resulting in increase in the survival time of cancer patients [10]. Different from normal cells, cancer cells rely too much on iron for cell proliferation [11]. This evidence suggested that ferroptosis has a different effect on cancer. In fact, targeting the tumor ferroptosis pathway is a new antitumor mechanism, which opens up a new therapeutic for the treatment of cancer [12].

Long-chain noncoding RNA (lncRNA) is an RNA with no or limited protein coding ability, whose length is about 200 bp to more than 100 kb [13]. lncRNAs participate in multiple biological regulatory processes, such as tumor occurrence, development, and metastasis [14]. One recent study revealed that lncRNA suppresses ferroptosis by acting as a competitive endogenous RNA (ceRNA) [15]. In a related study, lncRNA GABPB1 may be a key molecule for ferroptosis in hepatoma cells [16]. However, there are few studies to systematically evaluate the characteristics of ferroptosis-related lncRNAs and their relationship with the overall survival (OS) of CC patients.

In this research, we first established the prognostic multi-lncRNA signature of ferroptosis-related lncRNA based on Cancer Genome Atlas (TCGA) and FerrDb databases. Moreover, we discussed the effect of the novel ferroptosis-related lncRNA signature in immune response during CC prognosis. Our study provides a new gene signature for the prognosis prediction of CC patients and offers an important basis for the future study of the relationship between iron ferroptosis-related lncRNA and immunity in CC.

## 2. Methods

### 2.1. Collection and Preprocessing of Raw Data

The transcriptome profiling data including the RNA sequencing data of 309 samples (CC patients: 306; control groups: 3) and corresponding clinical data were obtained from the TCGA database (https://portal.gdc.cancer.gov/). The expression profiling matrix of both encoding gene and lncRNA was extracted with Perl. Ferroptosis-related genes were identified from the FerrDb database [17] (https://www.zhounan.org/ferrdb/). The clinicopathological data of CC patients were collected, including survival status, stage, TMN, grade, and survival time.

### 2.2. Data Processing of lncRNAs and Ferroptosis-Related Genes

The correlation test between ferroptosis-related mRNAs and lncRNAs was performed with Cor.test in R software (corFilter = 0.4; pvalueFilter = 0.01). Finally, the coexpression network of prognostic ferroptosis-related genes and lncRNAs was drawn by the Cytoscape software.

### 2.3. Construction of Prognostic Ferroptosis-Related lncRNAs Signature

We first used the survival “*R*” package (version: 3.2.1) for Cox regression analysis to construct survival prognostic characteristics. Then we selected lncRNA with significant statistical significance in univariate Cox regression for multivariate Cox regression. Finally, the risk score of patients was calculated according to the normalized expression level of each gene and the corresponding regression coefficient in the model. The formula = e^sum^^(each gene's expression×corresponding coefficient)^. CC patients were divided into the high-risk group and the low-risk group based on the median value of the risk scores.

### 2.4. Prognostic and Independent Analysis

We used Kaplan–Meier survival curves to distinguish the difference in overall survival (OS) between the different risk groups. In addition, we also used different R packages to construct K-M survival curve and analyze the ROC curve. Finally, we used the method of independent analysis to verify the independence of the model, such as stage and TNM.

### 2.5. The Predictive Nomogram

The gene set enrichment analysis (GSEA) was performed with GSEA 4.0.1 for investigating the potential mechanisms involved in the high-risk and low-risk groups. We considered *p* < 0.05 as statistically significant. We constructed a nomogram with prognostic characteristics to predict OS in CC patients at 1, 2, 3 and 5 years. Finally, the “prcomp” function of “stats” R package is used for principal component analysis (PCA).

### 2.6. Immunoinfiltration Analysis

We calculated the relative proportion of tumor infiltrating immune cells using the CIBERSORT algorithm to understand the infiltrating immune cells in the CC microenvironment associated with multi-lncRNA signature. Then we used the Wilcoxon test to compare the composition fraction of infiltrating immune cells between two different risk groups. Finally, we used Pearson correlation analysis to find out the relationship between lncRNA and significantly infiltrating immune cells.

### 2.7. Statistical Analysis

The R software was used for survival, Cox regression, and PCA analyses. We used the “survival R” and “surviviner R” packages for Kaplan–Meier analysis. Moreover, we validate the prediction model by using the “survival R,” “surviviner R,” “survival ROC R,” “pheatmap R,” and “ggpubr” software packages. GSEA was used to analyze the function of two risk groups of lncRNAs. When the *p* value <0.05, the difference was statistically significant.

## 3. Result

### 3.1. Identification of Ferroptosis-Related lncRNA in CC

**Figure 1:**
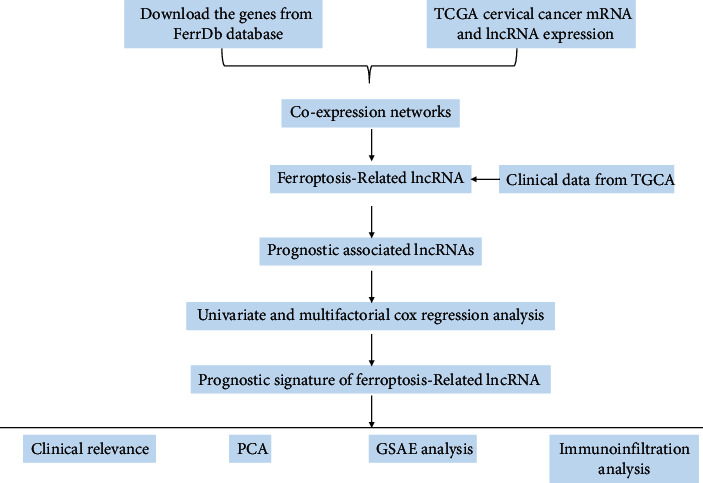
The flow-process diagram of the study.

**Figure 2:**
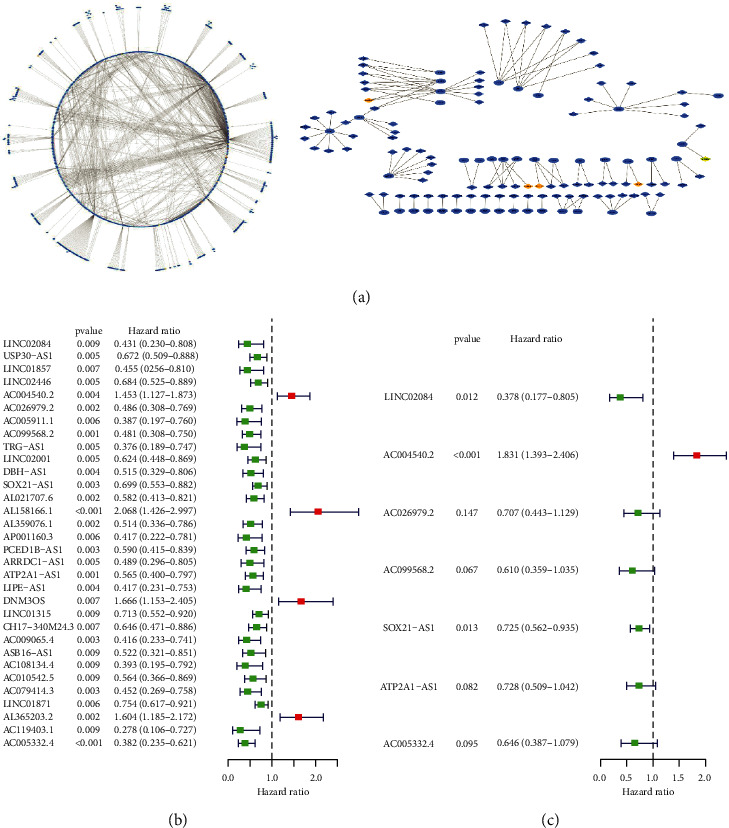
The coexpression network and Cox regression analysis result. (a) The network of ferroptosis genes and lncRNAs. (b) The forest plot of univariate Cox regression confirmed 32 ferroptosis-related lncRNAs. (c) The forest plot of multivariate Cox regression confirmed 7 ferroptosis-related lncRNAs.

Our flow-process diagram is shown in Figure 1. We constructed a coexpression network of lncRNA and ferroptosis-related genes through the “limma package” of R studio and Cytoscape 3.7.1 to obtain ferroptosis-related lncRNA (Figure 2(a)). The lncRNA whose expression level was significantly correlated with one or more of the 211 ferroptosis-related genes, with the correlation coefficient |*R*^2^| > 0.4 at *p* < 0.01, was considered a ferroptosis-related lncRNA. Finally, 1393 ferroptosis-related lncRNAs were identified, with 1346 ferroptosis-related lncRNAs positively correlated and 47 ferroptosis-related lncRNAs negatively correlated with CC.

### 3.2. Construction and Validation of the Ferroptosis-Related lncRNA Feature of CC

**Figure 3:**
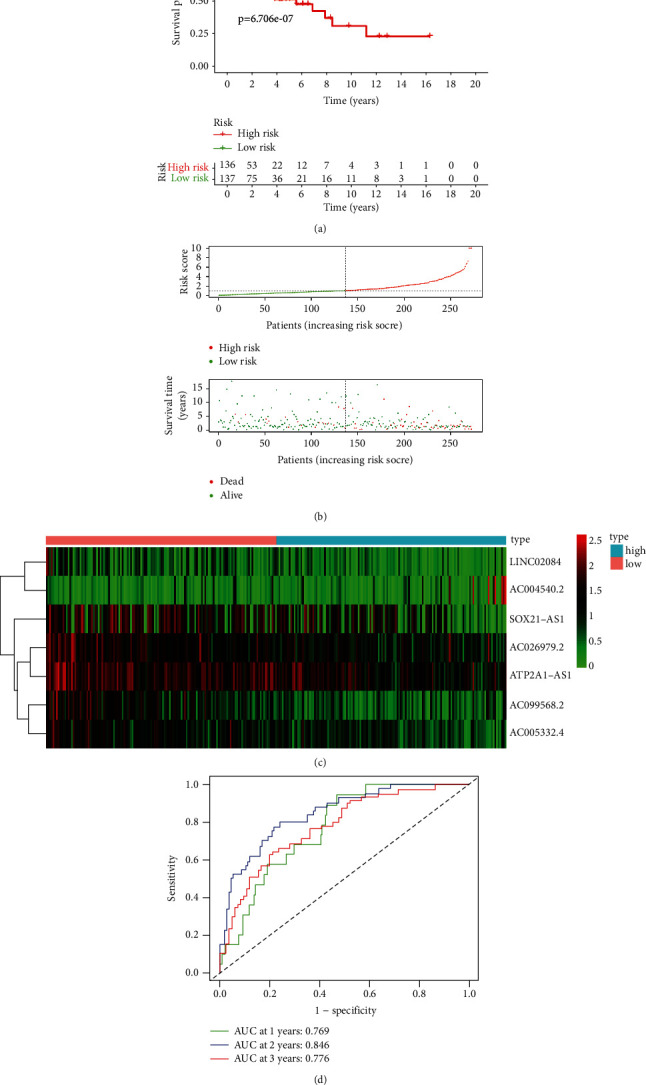
Prognostic analysis of the ferroptosis-related lncRNAs feature. (a) Kaplan–Meier curve of the patients in the high-risk and low-risk groups. (b) The rank of calculated risk score. (c) Heatmap of expression of 7 ferroptosis-related lncRNAs. (d) Time-dependent ROC curve analysis.

We first determine the prediction model based on univariate Cox regression analysis, and there were 32 lncRNAs related to ferroptosis in the prediction model (Figure 2(b)). Then these lncRNAs are included in the multivariate COX analysis (Figure 2(c)). Finally, there were 7 ferroptosis-related lncRNAs (LINC02084, AC004540.2, AC026979.2, AC099568.2, SOX21-AS1, ATP2A1-AS1, and AC005332.4) that can be considered an alone prognostic factor for CC. On the basis of the median risk score, all samples were allocated to a high-risk group (*n* = 136) and a low-risk group (*n* = 137). According to Kaplan–Meier analysis, poorer overall survival was associated with high-risk lncRNA expression (*p*=6.706*e* − 07, Figure 3(a)). The mortality of CC patients in the low-risk group was lower than that in the high-risk group (Figure 3(b)). The heatmap showed that the expression of lncRNAs (SOX21-AS1, AC026979.2, ATP2A1-AS1, AC099568.2, and AC005332.4) was significantly upregulated in the low-risk group, while the lncRNAs (LINC02084 and AC004540.2) were downregulated in the low-risk group compared to the high-risk group (Figure 3(c)). The predictive performance of OS risk score was evaluated by the time-dependent ROC curve, and the area under the curve (AUC) was 0.769 in 1 year, 0.849 in 2 years, and 0.776 in 3 years (Figure 3(d)). These results suggested that ferroptosis-related lncRNAs were a major risk factor for CC patients.

### 3.3. Independent Analysis of Prognostic Model and Other Clinical Variables

**Figure 4:**
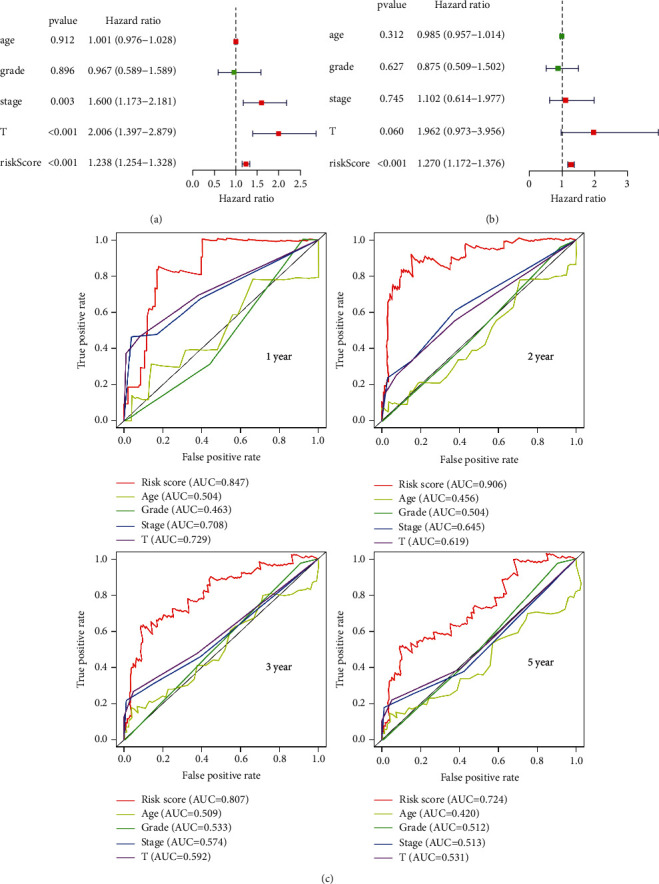
The clinicopathological characteristics. (a) The forest plot of univariate Cox regression. (b) The forest plot of multivariate Cox regression. (c) Multi-index ROC curve analysis compares the AUC value of the risk prognosis model and the clinical index prognosis model.

**Figure 5:**
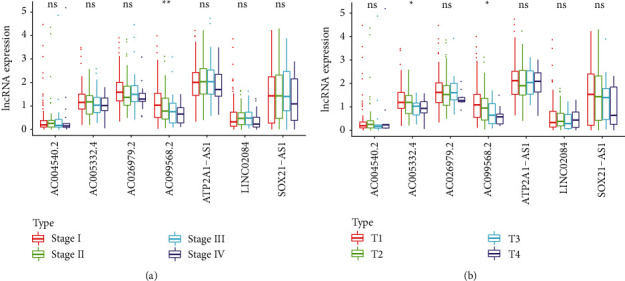
Independent prognostic value of the ferroptosis-related lncRNAs feature. (a) Stratification analyses of stage. (b) Stratification analyses of T stage.

Based on the predictive model, we used Cox regression to analyze the clinical feature of CC. There were significant differences in risk score, pathological T staging, and stage related to overall survival by univariate independent prognostic analysis (*p* < 0.05, Figure 4(a)). The risk score can be used as an independent forecast of CC in the multivariate Cox regression analysis (Figure 4(b)). Overall, the independent prognostic analysis of single factor and multiple factors showed that the predictive model is an independent predictive element. Multi-index ROC curve analysis compared the AUC values of the risk prognosis model and the clinical indicator prognosis model, which expressed that the AUC values of the risk score for 1-year, 2-year, 3-year, and 5-year survival are 0.847, 0.906, 0.807, and 0.724, respectively, and the areas are all maximum (Figure 4(c)). In addition, the hierarchical analysis was used to determine the independence of the prediction model (Figures 5(a) and 5(b)). For stage, AC099568.2 was significantly upregulated in the early stage of CC, whereas gradually downregulated as cancer metastasized (*p* < 0.001). For pathological T phase, AC005332.4 and AC099568.2 were statistically significant (*p* < 0.05), and the expression of AC099568.2 decreased with the progression of the T phase. These results revealed that the signature of ferroptosis-related lncRNAs can be used as a model for predicting CC.

### 3.4. Gene Set Enrichment Analyses

**Figure 6:**
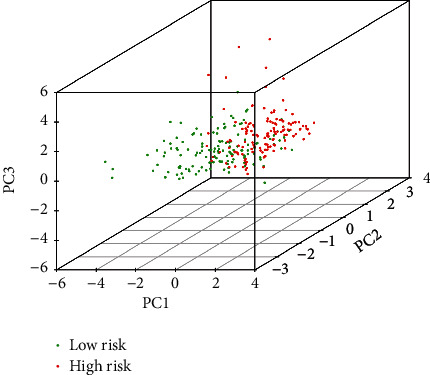
PCA.

**Figure 7:**
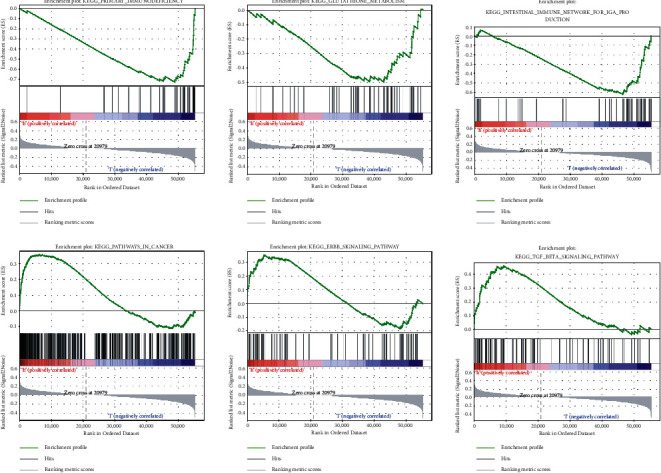
GSEA for the high-risk and low-risk groups.

To determine the difference between the diverse groups in lncRNA based on the model, we performed a PCA (Figure 6). Our results demonstrated that the two groups of patients spread in different directions. And the model lncRNAs divided CC patients into two specific parts, indicating that the prognostic status of CC patients in the two groups is very different. Furthermore, we performed GSEA on the two groups to find the possible biological function of the model of CC (Figure 7). GSEA revealed that ferroptosis-related lncRNA prognostic models mainly regulated immune- and cancer-related pathways, such as DNA replication, primary immunodeficiency, ERBB signaling pathway, pathways in cancer, the intestinal immune network for IGA production, and BETA signaling pathway. These results suggested that these related biological pathways play an important role in the carcinogenesis of CC.

### 3.5. The Immune Cell Infiltration Landscape in CC

**Figure 8:**
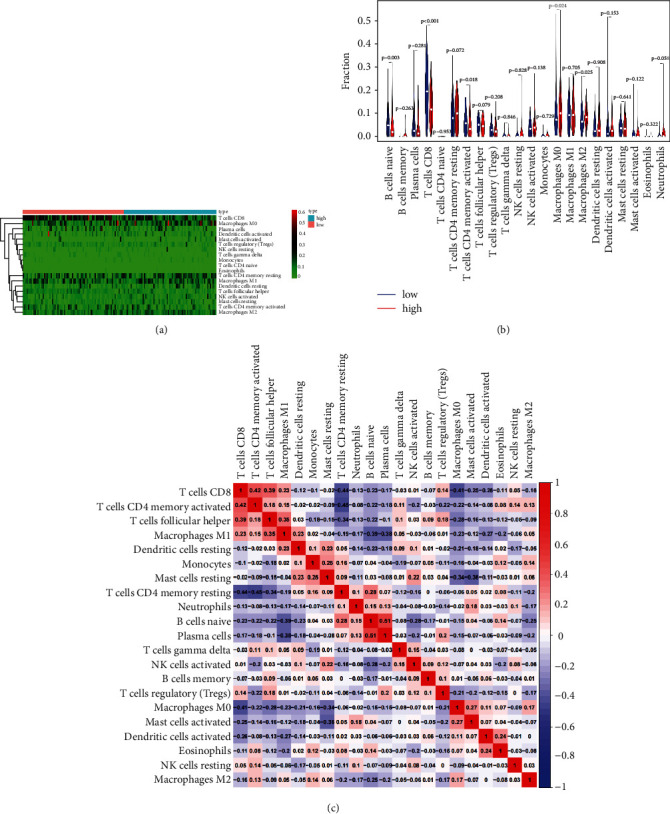
Comparison of 22 immune cell infiltration levels between the high-risk and low-risk groups. (a) Heatmap. (b) Violin plot. (c) Correlation matrix of immune cell proportions.

We used the CIBERSORT algorithm to analyze the connection between ferroptosis-related lncRNAs and antitumor immune. The results reasonably showed that there was a significant difference in the proportion of tumor infiltrating immune cells between the low- and high-risk groups (Figure 8(a)). We constructed a violin chart to compare the difference in immune cell infiltration between the low- and high-risk groups. The result displayed that there were significant differences in B cells native (*p*=0.003), T cells CD8 (*p* < 0.001), T cells CD4 memory activated (*p*=0.018), macrophage M0 (*p*=0.024), and macrophage M2 (*p*=0.025) between the two groups (Figure 8(b)). The correlation matrix of the proportion of all cancer infiltrating immune cells is shown in Figure 8(c). These results demonstrated that there were differences in immune-related genes between the high-risk group and the low-risk group, which may partly explain the significant difference in prognosis between subgroups.

## 4. Discussion

In the world, two-thirds of CC patients are still diagnosed as advanced. Although they have been treated with a variety of methods, they have lost the chance of radical cure [18]. In recent years, ferroptosis can help remove defective cells, which has become a new treatment method for tumors [19]. Moreover, lncRNAs have a profound influence in the occurrence and change of cancer [20]. Meanwhile, the importance of ferroptosis-related lncRNA in cancer development and treatment is increasingly recognized [21]. Zhou et al. certified that a risk model of ferroptosis-related lncRNA signature helped to predict immune infiltration, immunotherapeutic outcomes, and chemotherapy sensitivity in bladder cancer [22]. However, to our knowledge, there are few studies on the prognosis of ferroptosis-associated lncRNAs in CC. Therefore, in this study, we first constructed a coexpression network of lncRNA and ferroptosis-related genes and identified 1393 ferroptosis-related lncRNA. Then, we created a new prediction model integrating 7 ferroptosis-associated lncRNAs by univariate Cox regression and multivariate Cox analysis, which was then validated to perform well in an external dataset. The PCA result divided patients with different risk scores into two categories. The GSEA indicated that ferroptosis-associated lncRNAs regulated immune- and cancer-related pathways. Finally, the immune cell infiltration of the low-risk group and high-risk group was compared, high-risk group decreased levels of T cell CD8 and macrophage M2 and increased levels of B cells native, T cells CD4 memory activated, and macrophage M0 compared with the low-risk group.

Ferroptosis has been shown to be involved in cancer [23]. However, lncRNA may inhibit ferroptosis in cancer through the function of ceRNA [15]. In our research, we showed a coexpression network of ferroptosis-lncRNA, which proved that there is a regulatory relationship between lncRNA and ferroptosis-related genes. Combining this feature, we have built a prognostic model for CC. In our study, the K-M survival curve indicated that our prediction model is closely related to CC patients. In our results, the AUC values were 0.847 at 1 year, 0.906 at 2 years, 0.807 at 3 years, and 0.724 at 5 years. However, similar to this prognostic model, other researchers' AUC values were smaller than ours [24], which proved that the predictive ability of our model is relatively good. In addition, from the perspective of the cancer stage and pathological stage, AC099568.2 always was the most obvious lncRNA, indicating that the molecule is critical to the prognosis of CC. Ma et al. [25] reported that immune-related lncRNA signature was an independent prognostic factor for breast cancer and was closely related to clinicopathological features, indicating that this model is a very good prognostic tool for breast cancer. Moreover, similar to our research method, by constructing the prognostic 13-lncRNA signature of hepatocellular carcinoma and verifying it externally, it is proved that the model can be used to predict and diagnose the prognosis of hepatocellular carcinoma [26]. Compared with other studies [27], comprehensive analysis shows that our prognostic model is very reliable. These results manifested that the prognostic model can improve the prognostic ability of CC.

We have demonstrated that our model of ferroptosis-related lncRNAs can enhance the prognosis of CC. Next, we analyzed the biological functions of this prediction model through GSEA. GSEA revealed that ferroptosis-related lncRNAs were involved in the pathways of “primary immunodeficiency,” “DNA replication,” “ERBB signaling pathway,” “pathways in cancer,” “intestinal immune network for IGA production,” and “BETA signaling pathway.” DNA replication is crucial for tumorigenesis. Macheret and Halazonetis showed that DNA replication can drive cancer progression [28]. Moreover, Wang illustrated many cancers are related to overexpression or mutation of the ERBB receptor [29]. Low expression of ferroptosis-related genes is associated with poor prognosis of cancer and defective immune cell infiltration [30]. However, other studies reported that patients with high risk scores in model showed greater adaptive immunity [25]. In our study, the model showed “primary immunodeficiency,” which makes patients prone to frequent infection and malignant tumor [31]. Although the two models involve different functions, they all implied that these prognostic models had good predictive ability and played an important role in cancer immunotherapy. The above results proved that the predictive signature is related to not only tumorigenesis, but also correlative immune response.

Many studies have reported there is a relationship between lymphocyte infiltration and prognosis [32]. For example, the degree of lymphocytic infiltration in the primary tumor is positively correlated with the presence or absence of metastasis [33]. Therefore, we calculated the rate of immune cell infiltration in both risk groups. Different from the low-risk group, T cells CD8 and macrophage M2 cells were significantly reduced in the high-risk group. The main function of CD8 cells is to induce tumor cell death [34]. Besides, B cells native, T cells CD4 memory activated, and macrophage M0 cells were significantly increased in the high-risk group, which were generally used to defend against external aggressions [35]. Therefore, we considered that ferroptosis-related lncRNA is closely correlated with the proportion of tumor-infiltrating immunocytes in CC, and low-risk groups have more effective immune status than high-risk groups.

## 5. Conclusion

In summary, we have discovered a novel 7 ferroptosis-related lncRNA signature as a potential prognostic tool for CC patients. It is closely related to the tumor status, risk value, and OS. The signature offers a new insight into ferroptosis-related lncRNAs in CC and recognizes possible biomarkers for prognosis and immunological therapy.

## Data Availability

The data used to support the findings of this study are available from the corresponding author upon request.
